# Caring for a sick or injured child during the COVID‐19 pandemic lockdown in 2020 in the UK: An online survey of parents' experiences

**DOI:** 10.1111/hex.13347

**Published:** 2021-08-18

**Authors:** Sarah Neill, Rachel Carter, Ray Jones, Damian Roland, Natasha Bayes, Alison Tavaré, Joanne Hughes, Tracy Turner, Jade Chynoweth, Chantal Tan, Henriette Moll, Monica Lakhanpaul

**Affiliations:** ^1^ School of Nursing and Midwifery University of Plymouth Plymouth UK; ^2^ University Hospitals Leicester and University of Leicester Leicester UK; ^3^ Faculty of Health, Education and Society University of Northampton Northampton UK; ^4^ West of England Academic Health Science Network Bristol UK; ^5^ Mother's Instinct Support Group London UK; ^6^ Acutely Sick Kid Safety Netting Interventions for Families (ASK SNIFF) Parent Panel Northamptonshire UK; ^7^ Medical Statistics, Peninsula Medical School, Faculty of Health University of Plymouth Plymouth UK; ^8^ Erasmus MC‐Sophia, Department of General Paediatrics Rotterdam The Netherlands; ^9^ Policy & Practice Department, UCL GOS Institute of Child Health London UK

**Keywords:** children, COVID‐19, help‐seeking, pandemic, parents, RAG rating, SARS‐COV‐2

## Abstract

**Background:**

During the COVID‐19 pandemic, the first UK lockdown (March to May 2020) witnessed a dramatic reduction in children presenting to primary/emergency care, creating concern that fear of the virus was resulting in children presenting late.

**Methods:**

An online survey was co‐developed with UK parents to understand the impact of the lockdown on parents' help‐seeking for, and care of, their sick/injured child(ren). The survey was advertised through social media and snowballing to parents whose children had been ill/injured during the lockdown. Analysis used descriptive statistics, SPSSv25 and thematic analysis.

**Results:**

The survey was fully completed by 198 UK parents. The majority asked for help (144/198): from their family doctor (78), national helplines (48) or an Emergency Department (23). Most reported that their decision‐making had not changed, although how they sought help had changed. A few parents reported that the severity and duration of illness had increased because of uncertainty about and/or difficulty accessing services. Parents did not always report seeking help for symptoms rated red or amber by the Royal College of Paediatrics and Child Health. Parents reported accessing information through the internet or using information that they already had.

**Parent Contribution:**

This was a collaboration with parents from survey development to dissemination, with two parents being integral members of our research team.

**Conclusions:**

Our questionnaire was completed by parents who were not deterred from seeking help for their sick or injured children. Even for these parents, the lockdown changes to services created uncertainty about, and barriers to, accessing medical help for their children.

## 1 BACKGROUND

Before the coronavirus disease 2019 (COVID‐19) pandemic, significant numbers of children in the United Kingdom were being brought to primary and secondary care services with relatively low rates of admission.^1^, ^2^, ^3^ Given the low incidence of serious disease in children, policy makers and service providers felt that improvements could be made regarding parents' knowledge of childhood illness, when to seek help and how to care for their children with minor illness.^4^, ^5^, ^6^, ^7^, ^8^

During the first lockdown between March and May 2020 in the United Kingdom, when all nonessential shops and services including schools were closed, the numbers of children presenting to primary and emergency care fell significantly^9^, ^10^, ^11^, ^12^, ^13^ by up to 50% for all presenting conditions. While serious illness, such as sepsis, is relatively rare, this dramatic fall led to concern that children were not being brought to medical services and were potentially becoming more ill at home. These findings were supported by surveys of United Kingdom, Irish^12^ and Dutch^14^ paediatricians who were asked to report children presenting late to hospital. Before and during the pandemic, a small proportion of children were reported to present late in the course of an illness, as indicated by RAG (red, amber, green) symptoms of illness severity (based on the Royal College of Paediatrics and Child Health's [RCPCH] safety net tool^15^). RAG systems ascribe a relative risk to a collection of physiological and behavioural characteristics and assign a green (safe for discharge), amber (needs evaluation) and red (needs specialist input and treatment) label to them. Examples include the UK's National Institute for Health and Care Excellence (NICE) Feverish illness in Childhood (2019) and Sepsis guidelines (2017).

However, it remains unclear whether late presentations increased during the pandemic.^16^ Anxiety about using health services because of fear of infection, amongst the public, was reported during earlier epidemics: SARS (2003) in Canada^17^ and Taiwan,^18^ Ebola in West Africa (2014–2016)^19^ and H1N1 (2009) in Hong Kong^20^ and in Turkey.^21^ In the United Kingdom, it was assumed that it was the worry about becoming ill with COVID‐19 that was causing parents to keep their children at home when they were ill or injured.^22^ Messages from the UK government initially asked the public to avoid using health services unless it was really necessary. These messages included ‘*only call 111* [the National Health Service (NHS) telephone helpline NHS111] *if you're unable to get help online'*. Although this advice no longer appears on government websites, it continues to be repeated across UK regional health service webpages.^23^, ^24^, ^25^ This reduction in access to services may also have created positive outcomes for parent's self‐care of their children, as they may have developed ways of coping with, and managing, their child's illness or injury independently.

At the time of this survey, there was no evidence to explain why the numbers of children presenting to healthcare had fallen, nor was there any information about whether parents were using other health services instead or seeking help or information elsewhere. Understanding how parents seek help for sick or injured children during a pandemic is essential if services are going to be configured to support parents to ensure timely access to health services in the future. We designed the survey reported here to gather evidence directly from parents.

## 2 AIM

This study aimed to understand the impact of the first UK lockdown on parents' help‐seeking for, and care of, sick or injured children during the COVID‐19 pandemic.

## 3 RESEARCH QUESTIONS

- How did parents seek help for sick or injured children during the lockdown?
- How did parents care for sick or injured children during the lockdown?
- How did parents' help‐seeking for, and care of, sick or injured children change during the lockdown?
- Why did parents' help‐seeking behaviours for sick or injured children change during the lockdown?

## 4 METHODS

The study used a descriptive survey design to rapidly gather data from a large sample of parents while the first UK lockdown was still in place. This approach enabled data collection while parents' experience of managing a child with an illness or injury during lockdown was fresh in their minds. An online SNAP survey (www.snapsurveys.com) was chosen to enable anonymized data collection without the need for face‐to‐face contact, thus avoiding any additional risk of exposure to COVID‐19 infection. See Supporting Information Appendix S1 for the survey questions. Surveys have the advantage of facilitating the collection of data from a wide range of participants who are geographically disparate. The survey consisted of multiple‐choice questions with ‘other' options to add free text to explain alternative responses. Questions within the survey were designed using evidence from research exploring parents' usual care for sick or injured children at home and their decision‐making about seeking medical help for their children.^26^, ^27^, ^28^, ^29^, ^30^ Questions about the symptoms that their child had experienced were structured using the poster developed by the RCPCH to help parents know when to seek help for a sick or injured child.^15^ The phrase ‘Stay Home period’ was used in the survey to refer to lockdown as this was the term used by the UK government at the time. The resulting questionnaire was reviewed twice by a small group of parents (drawn from a parent panel and parent members of existing research teams) to establish face validity. Phrasing and sequencing of questions were changed following each parental review. The survey took approximately 5–10 min to complete. A ‘save and return later’ option was provided, bearing in mind parents' busy lives. As the aim of this survey was descriptive, a power calculation was not performed. However, for a sample size of *n* = 100, the 95% confidence interval for a 0.5 (50%) estimate of proportions is approximately 0.4–0.6 (40%–60%).^31^, ^32^

### 4.1 Parent contribution

Parents have been involved in the project as research team members and consequently have contributed to each stage of the work as their own lives permit. These parents reviewed the survey, helped to disseminate the survey through social media and commented on the write‐up of the findings. Having more than one parent in our research team ensured stable engagement from our parent collaborators. This was important because family life can be unpredictable as the needs of children change from moment to moment.

### 4.2 Ethical considerations

Participant information about the purpose and conduct of the research was provided at the beginning of the online survey, which concluded with a statement that choosing to complete and submit responses to the survey equated to giving consent to their responses being used in the project. The survey introduction also included a statement about the anonymity of responses (no personal identifiable data were collected) and the corresponding inability to withdraw responses once submitted. Information was also provided on how the data will be used and disseminated. Ethical approval for the project was granted by the University of Plymouth's Faculty of Health research ethics committee on 5 May 2020 (Ref 2020–2216). Data will be stored securely on the password‐protected University OneDrive for 10 years in accordance with University policy.

### 4.3 Study participants

We asked parents whose children aged under 18 years were ill or injured during the first pandemic lockdown living in the United Kingdom at the time to participate. We excluded those whose children had not been ill or injured and/or who were living outside the United Kingdom.

### 4.4 Recruitment

Parents were recruited through social media and snowballing. Information about the survey was posted on Twitter and Facebook and emailed to professional contacts (outside the NHS) with a request that colleagues share the information about the project with their contacts. The survey was also advertised on charity and other non‐governmental organizations' websites (*n* = 15) and professional organizations' (*n* = 33) websites. We used Google Docs to keep track of where survey information was shared. This method was selected following the success of a survey of children's and parents' access to information about COVID‐19 during the lockdown.^33^ A short introduction to the survey for use on social media was circulated to all our contacts; see Supporting Information Appendix S2. The survey was open to parents from 7 May to 21 June 2020, by which time lockdown in the United Kingdom had eased, with some children returning to school and nonessential shops reopening. Despite additional advertising, at this point, survey completion had also fallen, probably as the survey no longer seemed relevant to parents.

### 4.5 Data analysis

Statistical data were analysed using descriptive statistics and SPSSv25. The free text data were analysed thematically, drawing on Braun and Clarke's^34^ methodology to identify themes within the qualitative data. The level of severity of symptoms was identified using the RAG traffic lights coding in the RCPCH advice for parents.^15^ These RAG ratings were then used to explore how parents responded according to professional categorization of the severity of symptoms.

## 5 RESULTS

The survey was fully completed by 198 parents who reported that their child had been ill or injured during the lockdown; please see Supporting Information Appendix S3 for the numbers of completions in each week the survey was open. Incomplete returns (*n* = 204) were not included in the main analysis. However, of these 204 incomplete surveys, 53 completed the two main questions related to help‐seeking behaviour before lockdown and during lockdown; a breakdown of the completeness of surveys can be found in Supporting Information Appendix S4. This subset of 53 partial complete surveys was compared to the 198 completed surveys (Supporting Information Appendix S5). Completed surveys were fairly evenly divided between those concerning boys and girls (104/94; 52/48%). A quarter (25%) had a pre‐existing illness, most commonly atopic illness (asthma, eczema, allergy). The majority of returns concerned illness in the age group of 5–12 years; see Table 1 for details on the age of the children by presentation.

**Table 1:** Age of the children by presentation

Age of the child	Illness, *n* (%)	Injury, *n* (%)	Total, *n* (%)
Under 12 months old	11 (91.7)	1 (8.3)	12 (6.1)
12 months or over, but under 24 months old	12 (70.6)	5 (29.4)	17 (8.6)
2 years or over, but under 5 years old	26 (70.3)	11 (29.7)	37 (18.7)
5 years or over, but under 12 years old	70 (70.7)	29 (29.3)	99 (50.0)
12 years or over, but under 16 years old	19 (73.1)	7 (26.9)	26 (13.1)
16 or 17 years old	6 (85.7)	1 (14.3)	7 (3.5)
	144 (72.7)	54 (27.3)	198 (100.0)

Parents with children of primary school age (5–12 years) were more likely to fully complete the questionnaire, and those with children over 12 were more likely to drop out from the start (Supporting Information Appendix S6). Those with children less than 5 years tended to complete data on age and gender and then drop out. There was no difference by gender. The remaining results are for the 198 complete surveys only.

Most families (126/64%) selected urban as a description of the area where they lived; 72/37% chose rural as a description of the area where they lived. The largest group of respondents were from the South West of England (83/42%), although the sample did include parents from every area of the United Kingdom (see Supporting Information Appendix S7).

### 5.1 Symptoms reported by parents

Parents reported a wide range of symptoms in the categories provided (Table 2), and a further 73 signs and symptoms were added in the ‘Other’ category. These ‘Other’ symptoms included 19 gastrointestinal symptoms, 15 ‘cough’, 17 skin infections or inflammation, 3 dental problems, 3 foreign bodies, 4 sleepiness or fatigue, 3 mental health crises and one each of the following: allergic reaction, neck pain, hernia, shaking and loss of smell and taste. In each main symptom group, we identified those who also reported asking for help or not asking for help. The only significant difference between categories was for ‘Other’ symptoms—more ‘Other’ sought help than those with predefined symptoms (χ^2^ = 8.7, 1 *df*, *p* = .003); no correction was made for multiple tests. However, as this was a heterogeneous group, no clear conclusions can be drawn.

**Table 2:** Symptoms reported by parents (more than one answer possible)

Symptom group	Number of responses	Asked for help, *n* (%)	Did not ask for help, *n* (%)
Pain	107	73 (68.2)	34 (31.8)
Change in behaviour	58	42 (72.4)	16 (27.6)
Injury	54	43 (79.6)	11 (20.3)
Skin	39	33 (84.6)	6 (15.4)
Breathing	36	27 (75.0)	9 (25.0)
Dehydration	35	21 (60.0)	14 (40.0)
Temperature	26	19 (73.1)	7 (26.9)
Other	73	62 (84.9)	11 (15.1)

Each symptom contained a number of items reflecting those included in the RCPCH advice for parents^15^ within which symptoms are rated for severity using RAG ratings. The RAG rating for each item was retained in the analysis and an overall RAG rating was identified for each child (the highest RAG‐rated symptom reported by parents for the individual child; see Supporting Information Appendix S8 for examples of how this was worked out for individual children). This approach enabled us to map the RAG rating for the child against parents' reported help‐seeking; see Table 3.

**Table 3:** Overall RAG rating for each child and parents who reported help‐seeking

	Overall RAG rating for each child			
Parents reported help‐seeking	Green	Amber	Red	Total
Did you ask for medical help for your child for this illness/injury?, *n* (%)				
Yes	36 (72.0)	78 (71.6)	30 (76.9)	144
No	14 (28.0)	31 (28.4)	**9 (23.1)**	54
Total	50	109	39	198

*Note:* The bold numerals highlight the number of children with red RAG rated (serious) symptoms whose parents reported not seeking help.

Abbreviation: RAG, red, amber, green.

Worryingly, 9 parents' reported symptoms rated red (for further details, see Supporting Information Appendix S9a) and 31 reported amber‐rated symptoms for which they did not seek help. When asked what their usual response would be for this illness before the lockdown, 8 parents reporting red symptoms and 24 reporting amber symptoms said that they would normally care for their child at home. Interestingly, three parents who asked for help this time would not normally have done so and parents sought help for 36 children RAG‐rated green (see Supporting Information Appendix S9b).

### 5.2 Parent's reported sources of help for a sick or injured child during the lockdown

The majority of parents did ask for help for their child (144/198, 73%), most commonly from their General Practitioner (GP)/family doctor (78), NHS111/NHS24 national telephone helpline (48) or an Emergency Department (ED) (23). See Table 4 for all sources of help reported. The total number in the table exceeds the number seeking help as some people reported using more than one source of help.

**Table 4:** Sources of help used by parents

Source of help	Number of responses
Emergency department	23
Called 999	5
Children's Assessment Unit or Open access to children's ward	2
Dental services	2
GP out of hours	4
GP phone consultation	2
GP surgery	64
GP website	8
Health professional family member	1
Health visitor	1
Homeopath	1
NHS111/NHS24	48
Pharmacist	1
Specialist nursing or medical services	5
Urgent care or minor injuries service	18
Video consultation	5
Walk‐in centre	2

Abbreviation: GP, General Practitioner.

We asked parents to tell us more about their experiences of seeking help. These free text responses fell into two broad groupings: positive and negative experiences.

Positive experiences included receiving detailed advice that supported home care or provided information about what to do if their child deteriorated, GP consultation systems that facilitated assessment without face‐to‐face contact including video consultations, telephone consultations and the ability to send the GP photographs and helpful follow‐up calls. Phone consultations were reported to be quick and thorough. Parents found these systems reassuring. Parents also reported positive experiences when having to seek face‐to‐face help as they found that the protective systems in place reassured them that the risk of COVID‐19 infection in that health service was low.

Negative responses included fear of COVID‐19, which was reported by two parents to have resulted in up to 7 weeks' delay in seeking help, fear of attending face‐to‐face services and panic when having to attend. One parent reported that the GP they consulted appeared to be scared to conduct a physical examination. The appearance of professionals in full personal protective equipment increased anxiety for one parent. Concern about burdening the NHS appears to be secondary to worry about contracting the coronavirus. One area that was repeatedly reported to present difficulties was NHS111. Several parents reported difficulty getting through, one parent giving up and going to ED, long waits for call backs of up to 6 hours and call handlers treating every illness as COVID related.

The impact of the pandemic on the way in which services were delivered was also reported to impair access to services, resulting in delayed treatment, with a consequent increase in the duration and/or the severity of illness. For example, one child had an ear infection that was reported to progress to perforation as a consequence of not being able to access treatment earlier. Access to mental health services was also reported to be difficult, with a no face‐to‐face consultation rule resulting in increasing severity of mental illness. Access to emergency dental services was reported to be impossible by one parent, leaving their child in pain.

Mixed experiences were reported by several parents who found that communication between different parts of the health service was at times poor and/or inconsistent; at other points in the illness journey for their child, they reported receiving excellent, informative care. The result of these mixed experiences was increased uncertainty about their child's illness and treatment.

Overall, the response of services to the pandemic has brought about positive and negative changes. Positively, the increase in virtual consultations was welcomed by parents, whilst negatively, access to some parts of the health service was severely impaired.

### 5.3 Parents' self‐reported care of their sick or injured children during the lockdown

Responses to the question about what else, other than seeking help, parents did about their child's illness or injury revealed that 106 parents treated their child themselves, 90 parents waited to see if they got better, 76 parents looked for information on how to manage the illness or injury at home and 48 parents used information they already had.

Where parents responded that they cared for the child themselves, we asked about what they used to treat their child. Collated results of the responses in predefined categories and responses added in the ‘Other’ category are presented in Table 5. Paracetamol or ibuprofen was the most commonly reported home treatment. This may reflect the number of children who were reported to experience pain and/or cultural preference in the United Kingdom for antipyretics/analgesics as the first line of treatment at home.

**Table 5:** Self‐care by parents

Home treatment reported	Number of responses
Antipyretics/analgesics (paracetamol/ibuprofen)	115
Home care	
General illness or injury care (rest, cuddles, treats, etc.)	83
Injury care (icepack, cleansing, bandaging, etc.)	45
Prescribed medication (Inhalers, topical/oral steroids or antibiotics)	19
Over‐the‐counter remedies (Vicks/Karvol, antihistamines, topical creams, gripe water, ear drops, throat lozenges)	16
Home remedies (e.g., honey and lemon, steam inhalation)	6
Nursing/medical care (e.g., chest physio., suction, OCD therapy etc.)	4
Complementary medicine	3
Medication from overseas (Smecta, France)	1

### 5.4 Parents' information‐seeking

Most parents (122, 62%) used no sources of information, 32 parents (16%) used one source of information and 44 parents (22%) used more than one source of information. Parents who reported looking for information on how to manage the illness were asked about the source of this information. The most common choices were from the internet (39), including NHS Choices (33), NHS App (22), Google (17), GP website (6), other websites (7) and social media (1). Family (8), friends (6) and family health professionals (11) were also reported sources. Traditional media such as television, radio and print media were not reported to be used by any parents. Other things that parents reported using were peer‐reviewed literature (2) and the *Little Orange Book* from the North East of England (1).

Information that parents already had in the home was reported to be from a family health professional (13), family or friends (6) and internet sources (9). The ‘Other’ responses consisted of six parents who reported using information from previous contacts with health services, five parents who were health professionals and used their own professional knowledge, three parents who referred to information from a specific health professional, team or service and one parent who used information from health professional friends and NHS111.

Parents liked information that was clear and concise, explained the cause and management, provided reassurance and confirmed knowledge, treatment and the need for treatment. Parents also liked information that provided a perception of the availability of advice, if needed, and that it was safe to use services. Information was either verbal or written. Parents liked information that included the following:

- Safety netting information on what symptoms to look out for including symptoms of COVID‐19, what to avoid, normal ranges and how long to wait before seeking help.
- Symptom‐specific information such as fever, diarrhoea, vomiting, rashes, heatstroke symptoms, signs of appendicitis, etc.
- Information on how to care for their child.
- Information on treatment of their child's illness.

Unhelpful information was described as information that was too slow to access (NHS111/NHS24), not specific to the age of the child, vague, unclear, incomplete (on self‐isolation and COVID‐19 testing), confusing or conflicting. Conflicting information was reported to be scary, as was information on potential causes of an illness. The internet was unhelpful for some as it provided too much information. Social media was disliked for spreading gossip and rumours, while mainstream news media was reported to be ‘COVID scary,' with much speculation, reflecting the difficulties of living with uncertainty during the pandemic.

### 5.5 Information about the pandemic and when to use health services

We also asked parents about where they had seen information about the pandemic. The majority (156) had seen NHS sources of information or information from government sources (126). Other frequently reported sources were from family/friends on social media (52), experts on social media (44) and other online experts (33). Two parents specifically mentioned BBC News.

Parents reported seeing information about when to use NHS111 (158), GP services (136) and EDs (120), showing that although this messaging was reaching the majority of parents, there was a significant group who were less informed, adding to their uncertainty about where to seek help when their child was ill or injured. We also asked what new advice on health service use they had seen since the beginning of the lockdown. Many parents were aware of advice to stay at home, self‐isolate if you have symptoms and phone first before seeking any face‐to‐face access to services.

Free text replies revealed that parents were aware of advice to either try not to call NHS111 in the early stages of the lockdown or to only call NHS111 with severe symptoms of COVID‐19 to later advice to call NHS111 first before calling the GP or attending ED. Parents also reported awareness of advice not to attend GP surgery or ED unless it is an emergency. Parents reported that they had heard that walk‐in centres, dentists and Child and Adolescent Mental Health Services (CAMHS) were all closed. Later on during the lockdown, parents reported having seen TV adverts reassuring people to continue to use the NHS, specifically not to hesitate to take a child to ED, which parents commented they had not seen before the pandemic. Appointments were reported to be replaced by phone calls, video calls, texts and emails. However, there were some free text replies from parents who were not sure, thought there were no changes or who found information unclear, confusing and were uncertain about what to do.

### 5.6 Impact of the changes to health services during the lockdown

The majority of parents (150, 75%) reported that the changes to health services had not affected the severity of their child's illness. Other parents thought that it had affected the severity of their child's illness (26), may have affected it (13) or did not know (9). Free text responses explained that, where there was an impact, it was related to the lack of a physical examination of their child and lack of access to, or delayed, investigations resulting in misdiagnoses (and wrong treatment), as it took longer to diagnose the illness and to obtain treatment. The consequences of these delays were reported to be more serious illness (physical and mental), longer duration of illness and slower recovery. Some parents also reported remaining uncertain about the nature of their child's illness.

More parents (42%) reported an impact on their child's treatment (51 yes/32 may be) rather than on the severity of their child's illness. Parents' free text responses included the following explanations for this impact. Cognitive behavioural therapy, operations and investigations were cancelled, as were routine treatment reviews for those with long‐term health needs, routine immunizations and developmental checks. One parent also reported that the type of surgery changed to a more invasive form (open rather than keyhole). All of these will have effects in the longer term beyond the pandemic. Virtual assessment was reported to lead to incorrect treatment and possible overuse of antibiotics. One parent reported reducing the dosage of paracetamol administered to a child so that their stock would last to the end of their self‐isolation period. Several parents reported the lack of access to physiotherapy services as it is ‘hard to do physio over the phone’. One parent expressed that the loss of this service may have long‐term effects on the health of the child concerned. Positively, another parent reported that, when they did need to use the ED, they were treated quickly and effectively as the department was very quiet.

### 5.7 How did parents' help‐seeking for, and care of, a sick or injured child change during the lockdown?

We asked parents whether or not they sought help for this episode of illness or injury in their child and what their usual response to this illness or injury would have been before the lockdown and compared their responses; see Table 6.

**Table 6:** Comparison of help‐seeking before and during the lockdown

Did you ask for help (in lockdown), *n* (%)	Would you *normally* have asked for help		
	Yes	No	Total
Yes	130 (67)	13 (6.7)	143 (73.7)
No	25 (12.9)	26 (13.4)	51 (26.3)
Total	155 (79.9)	39 (20.1)	194 (100.0)

*Note: N* = 194 [excludes 4 (2.0%) people who did not know or who would do ‘Other’].

Given the anxiety expressed by many of the parents, it was surprising to find that most parents did not report changing their decision to seek help during the lockdown. Although 25 parents (13%) who said they would normally seek help did not do so during lockdown, a smaller group (13/7%) reported seeking help who would not normally have done so.

When we compared help‐seeking for children with illness compared to help‐seeking for injury, we found no significant difference (2 × 2 cross tabulation: χ^2^ = 1.78, 1 *df*, *p* = .18).

There were differences in the places that parents reported seeking help; see Table 7. Some of these can be explained as responses to the advice to Stay Home unless it is an emergency, such as an increase in the use of the NHS111 telephone advice line, video consultations and use of the GP website. Numbers reported to attend some face‐to‐face services fell (minor injuries unit/walk‐in centre, GP surgery), but more parents reported using ED than they said they normally would.

**Table 7:** Where parents reported seeking medical help for their child's illness or injury

	Normally	Lockdown
Emergency department	11	23
Called 999	5	5
GP out of hours	4	4
Minor injuries unit	20	12
Urgent care centre	1	5
Walk‐in centre	4	2
Video consultation	0	5
GP website	1	8
GP surgery	82	64
NHS direct/NHS 111/NHS 24	20	48
Somewhere else	9	16
Total asked for help	155	143
Did not ask for help	43	54

Abbreviations: GP, General Practitioner; NHS, National Health Service.

### 5.8 Why did parents' help‐seeking behaviours for a sick or injured child change during the lockdown?

Parents who reported not seeking help during the lockdown were asked why they did not seek help. This was a multiple‐choice question to which parents responded as follows: 27 were not sure their child was ill or injured enough; 19 were worried about catching COVID‐19; 18 were worried about using a service needed by other people; 9 were worried about being criticized for using services if it was not an emergency; 8 were worried about it being busy and having to wait a long time; 4 parents reported that they did not have anyone to look after other children; and 1 parent reported that they did not have a car and did not want to use public transport. In the accompanying free text responses, some of the parents mentioned that their children's illnesses spontaneously resolved. Parents reiterated their fear of COVID‐19 and consequently of using services, and their confusion about where and when to seek help. Some parents also mentioned their need for information and equipment to be able to monitor their child at home when access to services was limited.

## 6 DISCUSSION

We aimed to understand the impact of the first lockdown in the United Kingdom on parents' help‐seeking for, and care of, a sick or injured child because the reduction in children seen by health services had raised concerns that parents were avoiding seeking help for their children because of the fear of COVID‐19. Parents did report worries about contracting COVID‐19; however, this anxiety did not appear to deter the majority of parents (73%) responding to this survey from seeking medical help for their child. There was a group of 25 parents (13%) who said that they would normally seek help, but did not do so during lockdown. Like the parents in Nicholson et al.'s^35^ survey, parents may have been more anxious about contact with health services. A small group of parents (7%) sought help who said they would not normally have done so. This may reflect the impact of uncertainty about access to services on parents' help‐seeking behaviour reported in earlier research.^36^, ^37^ There were some changes to the places that parents sought help, with the move to more telephone and video consultations. Surprisingly, more parents reported use of the ED than said they normally would have done for that illness/injury, contradicting results from attendance data that reported a reduction in ED attendance during lockdown in the United Kingdom and Italy.^9^, ^10^, ^11^, ^12^, ^13^ However, this was a relatively small sample; consequently, the findings have limited generalizability. Of course, the overall incidence of illness or injury in children everywhere, during lockdown, may have been much lower than in normal circumstances. Children's exposure to all infections was reduced by social distancing measures and their restricted environments also reduced accidental injuries.^22^

A few parents reported that changes to services, especially the reduction in face‐to‐face consultations and the cancellation of some services, led to lack of access to, or delay accessing, healthcare, providing another explanation for the reduction in child consultations. National data on antibiotic prescribing showed a significant reduction during the first lockdown, particularly in the South West,^38^ which may indicate a reduction in access to prescribing services and/or a reduction in infections in children and, in the South West, a reduction in the tourist population.

Most parents reported providing some care to their children independently at home, most commonly paracetamol or ibuprofen, which may be related to the high number of children for whom pain was reported as a symptom. The next most common symptoms reported were behavioural changes and injury, with symptoms of acute illness reported much less often, possibly also reflecting the reduction in all infections. Analysis of the professional severity scoring (RAG rating) of the symptoms reported highlighted a small group of parents who did not seek help for red or amber symptoms. Few parents reported using information already present in the home to help them manage their child's illness/injury. Most looked online for information, which can be scary, inconsistent or confusing.^8^ Together, these results indicate that safety netting information, to help parents determine the severity of their child's illness and whether or not they need to seek medical help, is still not easily available in a reliable form for parents, repeating earlier findings.^8^, ^39^ Lim et al.^40^ trialled a leaflet on the recognition of serious illness in children during the lockdown in the North East of England and found that parents felt it increased their confidence, although few had used the leaflet for a sick child. Mobile apps available in this area were not referred to by parents completing our survey.

### 6.1 Strengths and limitations

This was the first detailed survey that aimed to establish how the first UK lockdown during the pandemic influenced parents help‐seeking for, and care of, ill or injured children. We involved parents at every stage from design of the questionnaire to editing this paper. Patient and public involvement is considered to result in better‐quality research,^41^, ^42^ although there is no evidence concerning the most effective methods for doing so in child health research.^41^ Multiple‐choice questions provided a picture of responses for the whole sample, which were then augmented by the detailed free text responses.

The results included data from all four countries of the United Kingdom. However, far more people completed the question from the South West, illustrating the power of personal networks in engaging people in research, as the project leads were based in the South West. The sample included families living in both urban and rural settings and children across the childhood age range. However, the biggest group of returns was from parents of children aged 5–12 years. This does not reflect the age group with the highest incidence of childhood illness/injury or use of health services prepandemic,^3^, ^43^ but *may* represent the group whose parents were able to find time to complete the questionnaire. Alternatively, the reduction in infections may have been greater in this younger group of children. Public health services for the younger age group (0–2 years) were reduced/suspended^44^; consequently, this group of parents may have changed their help‐seeking during the lockdown as they were unable to access health visitors (UK public health nurses). No data were gathered to indicate how help‐seeking for this group of parents changed.

Use of symptoms drawn from RCPCH (2020) advice for parents enabled categorization of the severity of symptoms reported. This was also a limitation as it did not include symptoms that are known to cause parents to worry, such as cough, gastrointestinal symptoms or skin infections (commonly seen in primary care) that appeared in free text comments.

We did not collect information on ethnicity or socioeconomic status to maximize the number of returns, as we were not asking parents for detailed personal data. However, we did ask about the type of area where families lived and about their access to digital technology.

More parents might have completed the survey had there been funding available to advertise it or provide participating parents with incentives as illustrated by the survey conducted in Ireland.^35^ Lack of funding limited survey advertising to our networks and social media; consequently, we could not reach those without internet connections within more marginalized populations, creating a sampling bias. Given the association between digital exclusion and poverty,^45^ it is likely that we have not included the most disadvantaged families in our society whose health is poorer.^46^, ^47^ Had we have been able to capture data from these families, the results may have painted a different picture. We had a large number of people who did not complete the whole survey and so were not included in the main analysis. There is evidence that they were more likely to have been dissuaded from seeking help during the pandemic than those who fully completed the survey. We might extrapolate and assume that those who never started the survey were also more likely to have reduced help‐seeking behaviour.^48^, ^49^

## 7 CONCLUSIONS

The findings presented here show that in our sample, most parents were not deterred from seeking help for their sick or injured children. Instead, it was the changes to services during the lockdown that created uncertainty about, and barriers to, accessing medical assessment and treatment for their children. Together with findings showing that parents may not recognize potentially serious symptoms in their children, this indicated a need for easy access reliable safety netting information including contemporaneous information on local services.

When access to services is limited and parents have to monitor their children at home independently, parents also need equipment to assess their child's symptoms and a mechanism for communicating these symptoms to health professionals.

At the time of writing, the UK was in the midst of a third lockdown (January 2021). This highlights the importance of these findings being used to inform the development of interventions to improve parents' ability to determine when they need to seek help for a sick or injured child and support their access to services. Such resources will also help parents recognize the symptoms of COVID‐19. Continued access to services, including the ability for clinicians to visually assess children, is also crucial to prevent an increase in the numbers of children presenting late with more serious illness.

## CONFLICT OF INTERESTS

The authors declare that there are no conflict of interests.

## AUTHOR CONTRIBUTIONS

The original idea for the project came from Rachel Carter, and the project was led by Sarah Neill and Rachel Carter. The survey was designed by Sarah Neill and Rachel Carter. Joanne Hughes, Tracy Turner, Ray Jones, Damian Roland, Natasha Bayes, Alison Tavaré and Monica Lakhanpaul reviewed the survey, which was then revised by Sarah Neill. Ray Jones, Sarah Neill, Rachel Carter and Jade Chynoweth analysed the data. Sarah Neill wrote the first draft of the paper; all authors reviewed it and Sarah Neill revised it accordingly.

## Supporting information

Supporting information.

## Data Availability

Data available on request from the authors.
